# Supplementary material for: Characteristics and Electronic Band Alignment of a Transparent p-CuI/n-SiZnSnO Heterojunction Diode with a High Rectification Ratio
Source: Nanomaterials (Basel). 2021 May 7;11(5):1237. doi: 10.3390/nano11051237 (PMC8151173; doi:10.3390/nano11051237)
Supplement: Supplementary file 1 [file nanomaterials-11-01237-s001.zip › nanomaterials-1171308-supplementary.pdf]

nanomaterials

Supplementary Material

Characteristics and Electronic Band Alignment of a Transparent *p*-CuI/*n*-SiZnSnO Heterojunction Diode with a High Rectification Ratio

Jeong Hyuk Lee [1], Byeong Hyeon Lee [2], Jeonghun Kang [1], Mangesh Diware [1], Kiseok Jeon [3,4], Chaehwan Jeong [4], Sang Yeol Lee [5,*] and Kee Hoon Kim [1,6*]

[1]Center for Novel States of Complex Materials Research, Department of Physics and Astronomy, Seoul National University, Seoul, 08826, Republic of Korea
[2]Department of Microdevice Engineering, Korea University, Seoul, 02841, Republic of Korea
[3]Department of Chemical and Biomolecular Engineering, Yonsei University, Seoul 03722, Republic of Korea
[4]Smart Energy and Nano Photonics R&D Group, Korea Institute of Industrial Technology, Gwangju, 61012, Republic of Korea
[5]Department of Electronic Engineering, Gachon University, Gyeonggi-do, 13120, Republic of Korea
[6]Institute of Applied Physics, Department of Physics and Astronomy, Seoul National University, Seoul, 08826, Republic of Korea
* Correspondence: Correspondence: sylee2020@gachon.ac.kr (S.Y.L.); khkim@phya.snu.ac.kr (K.H.K.)

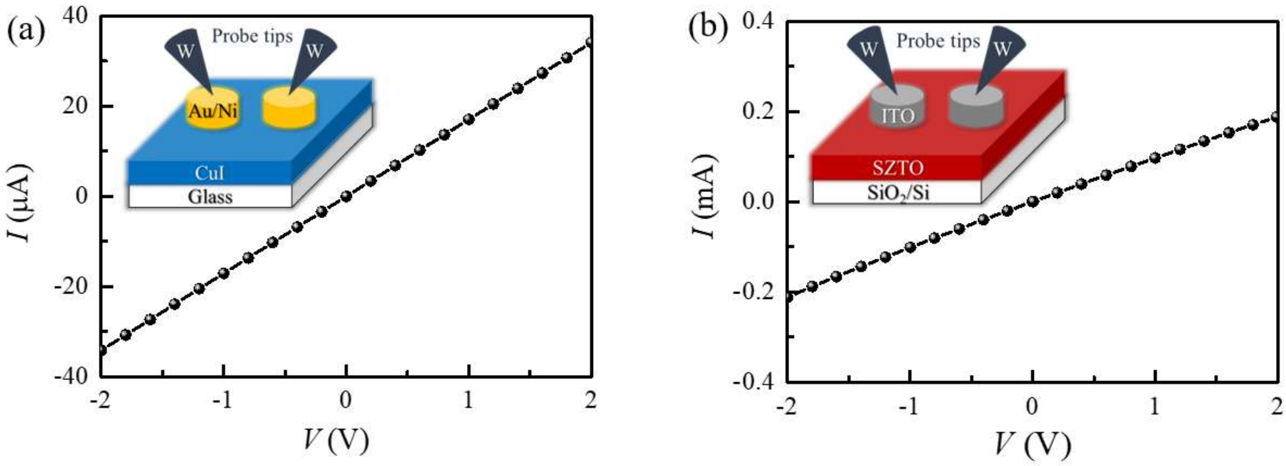

Figure S1. Current-voltage (*I-V*) characteristics of (a) Au/Ni/CuI and (b) ITO/SZTO/SiO_2/Si. The *I-V* curve measurements were carried out at room temperature without light exposure. Schematic drawings for the measurement configuration of the two-point contact method are presented in the inset.

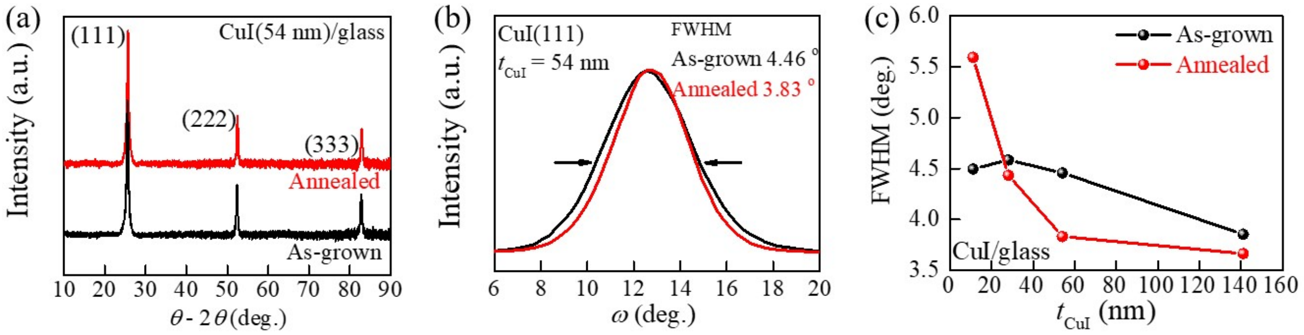

Figure S2. Before (black) and after (red) thermal annealing at N_2 atmosphere (a) X-ray θ-2θ scan profiles of the CuI(54 nm)/glass, exhibiting only (*l l l*) (*l* =1, 2, and 3) peaks. This indicates that the CuI film has grown with preferential alignment along the <111> direction. (b) The rocking curves of the CuI(54 nm)/glass with the full width at half maximum (FWHM) values as estimated from the CuI(111) peak. (c) The FWHM values of the CuI/glass as a function of the thickness of the CuI film (t_{CuI}).

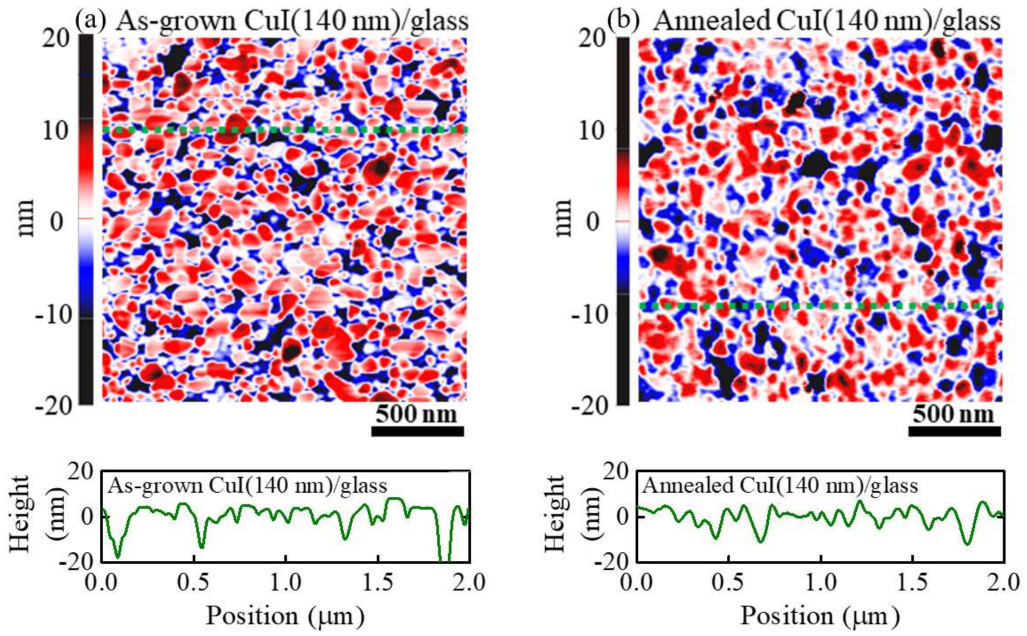

Figure S3. Surface topography images measured by atomic force microscopy (AFM) in a contact mode for (a) the as-grown CuI(140 nm)/glass sample and (b) the annealed CuI(140 nm)/glass sample. The bottom panels show line profiles along the green dotted lines shown in the AFM images. After thermal annealing, root-mean-square surface roughness (R_{rms}) in the whole area of $2 \times 2\ \mu m^2$ decreases from 5.46 nm to 4.03 nm and the averaged grain size increases from ~78 nm to ~82 nm.

In the AFM image of the as-grown CuI film (Fig. S3 (a)), each grain is represented by the red colored region while the grain boundaries are shown as white colored lines. In the annealed CuI film, the averaged grain size is about 82 nm, which is slightly increased from that of the as-grown film, ~78 nm; moreover, root-mean-square surface roughness (R_{rms}) in the whole area of $2 \times 2\ \mu m^2$ decreases from 5.46 nm to 4.03 nm. After the annealing, most of grains are better connected with the nearest neighboring grains so that the white borderline representing the grain boundary becomes unclear, indicating the merging of the grains. As a net effect, grain boundaries are indeed decreased after the thermal annealing. Thus, based on the experimental observation of the decrease of R_{rms} and the merged grains in the AFM images, we confirm that thermal annealing effectively reduces the grain boundaries. It is noted that the lateral and height resolution of the AFM images are nearly equal since the exactly same condition was used for the AFM measurements.

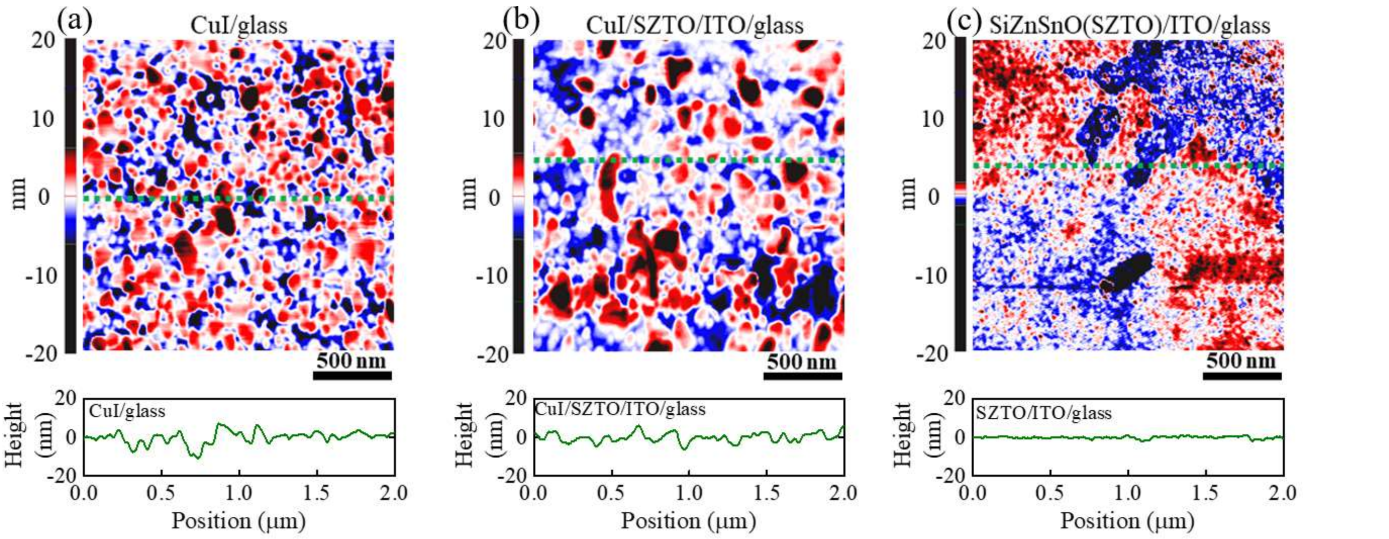

Figure S4. Surface topography images measured by atomic force microscopy (AFM) in a contact mode for the samples of (a) CuI(54 nm)/glass, (b) CuI(110 nm)/SZTO/ITO/glass, and (c) SZTO/ITO/glass. The bottom panels show the line profiles along the green dotted lines of each AFM images.

The root-mean-square surface roughness (R_{rms}) of a polycrystalline *p*-CuI film/glass is ~3.11 nm in the measured area of 2 × 2 μm^2. The CuI film grown on top of SZTO/ITO/glass shows R_{rms} = ~2.80 nm, which is similar to that of the CuI/glass (3.11 nm), implying that the surface topography of the *p*-CuI/*n*-SZTO heterojunction is nearly maintained in the *p*-CuI film, regardless of the bottom layers and the thickness of CuI, t_{CuI} = 54 or 110 nm. An amorphous SZTO film exhibits a smooth surface with R_{rms} = ~0.75 nm.

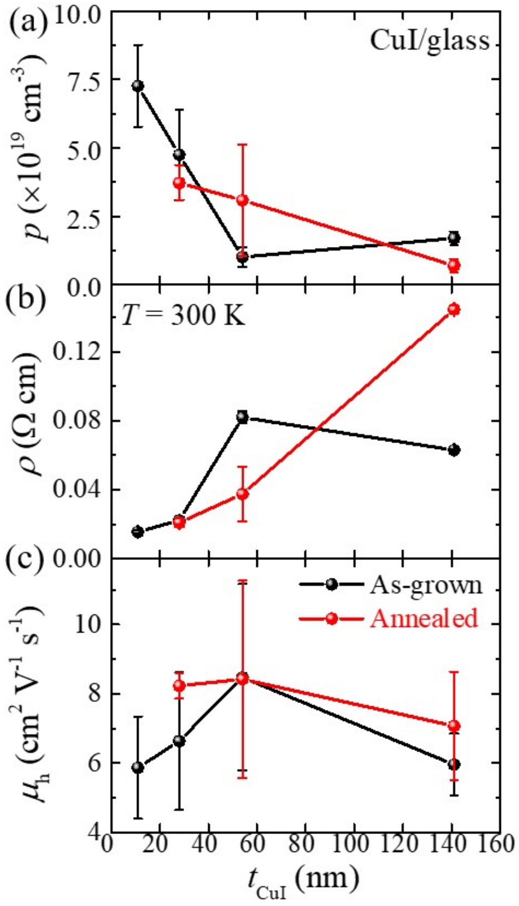

Figure S5. (a) Room temperature hole carrier density p, (b) electrical resistivity ρ, and (c) hole mobility μ_h as a function of t_{CuI}. The black and red colors represent the data of the as-grown and annealed CuI films on glass substrates, respectively. In the case of annealed CuI(11 nm)/glass, Hall resistivity could not be measured due to the high resistance of the CuI incurred during the thermal annealing. All the films show a positive Hall coefficient, indicating dominant p-type carrier. Hall effect measurements were carried out by the Van der Pauw method at room temperature without light exposure.

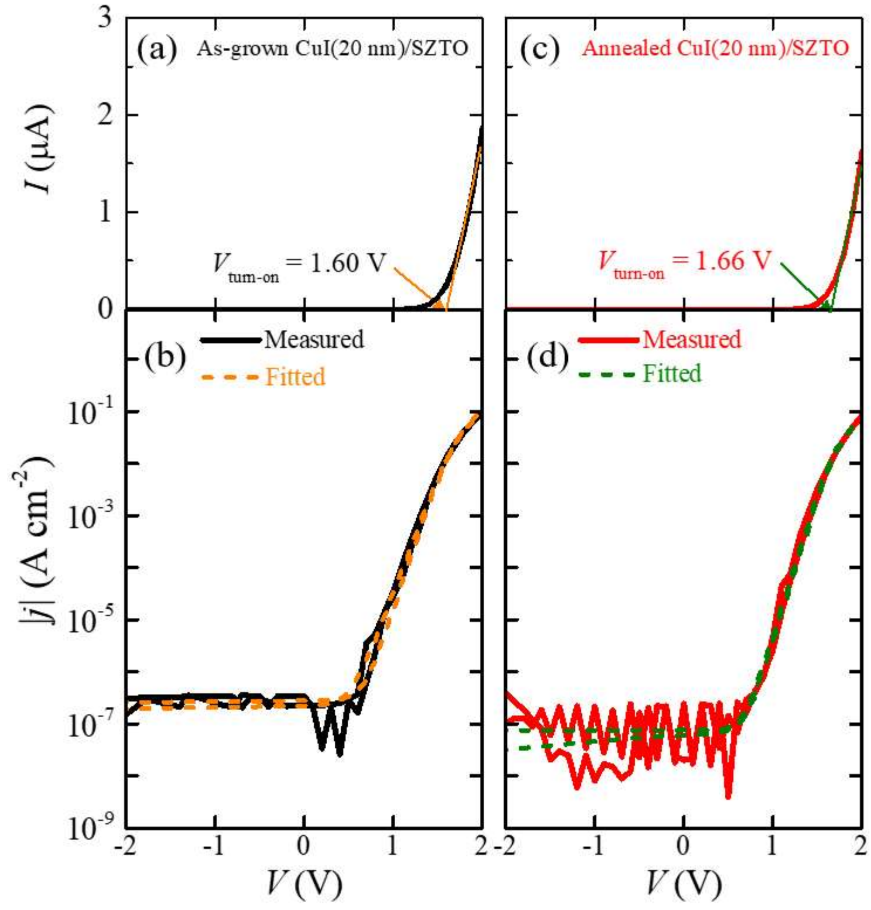

Figure S6. (a) The current-voltage *I*-*V* curve in a linear scale and (b) the current density-voltage |*j*|-*V* curve in a semi-log scale of the as-grown CuI(20 nm)/SZTO diode. (c) The *I*-*V* and (d) |*j*|-*V* curves of the annealed CuI(20 nm)/SZTO diode. The orange and green solid lines in the *I*-*V* curves represent the linear extrapolation to determine the turn-on voltage of a diode, $V_{turn\text{-}on}$, while the orange and green dashed lines in the |*j*|-*V* curves correspond to the diode fitting results based on Eq. (1).
